# Coffee reduces the risk of hepatocellular carcinoma probably through inhibition of NLRP3 inflammasome activation by caffeine

**DOI:** 10.3389/fonc.2022.1029491

**Published:** 2022-10-18

**Authors:** Frank S. Fan

**Affiliations:** Section of Hematology and Oncology, Department of Medicine, Ministry of Health and Welfare Taitung Hospital, Taitung County, Taiwan

**Keywords:** caffeine, coffee, hepatocellular carcinoma, NLRP3 inflammasome, pyroptosis

## Introduction

Hepatocellular carcinoma (HCC), a leading cause of cancer-related death in the world, was assessed to have a global incidence rate of 9.3 per 100,000 person-years with an approximately equivalent mortality rate of 8.5 in 2018 with substantial variations in different areas (1). The main risk factors for HCC have been attributed to chronic hepatitis B virus (HBV) and hepatitis C virus (HCV) infection, alcoholic cirrhosis, non-alcoholic fatty liver disease (NAFLD), alfatoxin, and aristolochic acid (2). Despite extensive vaccination for HBV and aggressive administration of anti-viral treatments against HBV and HCV, the International Agency for Research on Cancer reckons that 1,436,744 new patients will be diagnosed to have liver and intrahepatic bile duct cancer in 2040 on the basis of annual projections (3) with 75%-85% as HCC and 10%-15% as intrahepatic cholangiocarcinoma (4). Prevention of HCC and reduction of its death rate therefore become an urgent need in public health.

Interestingly, a recent dose-response meta-analysis disclosed that an extra two-cups of coffee per day reduced the risk of HCC by 35% without significant intervention from liver disease stage, body mass index, alcohol drinking, and viral hepatitis B or C infection (5). The inverse correlation between coffee consumption and HCC risk was shortly supported by another similar dose-response study reporting a 15% reduction in HCC risk by one cup of coffee per day (6). Following these, in a succeeding umbrella review of meta-analyses of observational studies accommodating 36 summary associations for 26 cancer sites, highly suggestive evidence was shown for an inverted relationship between coffee intake and the risk of HCC (7). However, the potential mechanism of coffee’s beneficial effect on reducing HCC development remains to be explicated and requires resolution in spite of thoroughly reviewing relevant literatures (8). Herein, an attempt to explain the causality will be made based on recent advancement of research on inflammasomes, the supramolecular cytoplasmic structures which drive immune responses and inflammation.

## The role of inflammation in hepatocarcinogenesis

Regardless of multiple aetiologies for HCC, progressive accumulation of molecular genetic alterations begins in preneoplastic dysplastic nodules resulting from aberrant hepatocyte regeneration (9). Prior to the formation of dysplastic nodules and cirrhosis, the liver has sustained chronic inflammation stimulated by endoplasmic reticulum (ER) stress induced by a variety of pathogenic factors (10). Among them, alcoholic steatohepatitis (ASH), NAFLD, chronic viral hepatitis B and C are most notable ones in which HCC always develops as a final outcome from a progressive process through inflammation to fibrosis and subsequent cirrhosis (11). In addition to ER stress, inflammasomes activation elicited by various pathogen- and danger-associated molecular patterns (12) might also play essential roles in generating inflammatory signalling cascades in inflammation-based hepatocarcinogenesis. The interaction between ER stress and inflammasomes is complex. Accumulating evidence shows that ER stress probably exerts its function through activating inflammasomes (13, 14) and promoting inflammatory process induced by inflammasomes (15).

Inflammasomes, the dominant pathway of inflammation and the pro-caspase-1 activating platforms, are cytoplasmic organelles containing one of the five pattern-recognition receptors, that is, the nucleotide-binding oligomerization domain (NOD), leucine-rich repeat-containing proteins family members NLRP1, NLRP3, and NLRC4, as well as absent-in-melanoma 2 (AIM2) and pyrin (16). Activation of inflammasomes leads to augmentation of caspases, resulting in apoptosis, pyroptosis, release of proinflammatory cytokine interleukin-1β (IL-1β) and interleukin-18 (IL-18) (17). The NLRP3 inflammasome which comprises NOD-like receptor family pyrin domain-containing 3 (NLRP3), apoptosis-associated speck-like protein containing a caspase recruitment domain (ASC) and caspase-1 cooperates with ER stress and mitochondria damage in the pathogenesis of many liver disorders such as ASH, NAFLD, nonalcoholic steatohepatitis (NASH), hepatic ischemic injury, and hepatotoxicity (18, 19). Moreover, experimental research has demonstrated that deubiquitination and activation of NLRP3 inflammasome facilitates HCV life cycle in HCV-infected hepatic cells (20) and HBV X antigen activates NLRP3 inflammasome under oxidative stress and advances pyroptosis in hepatic cells (21). Notably, the pyroptosis mediated by inflammasomes also takes part in the pathogenesis of chronic virus infection and liver fibrosis, affecting hepatocytes, Kupffer cells, hepatic stellate cells, and inflammatory cells as a whole (22).

Therefore, inhibition of inflammasomes and the resulting pyoptosis has become hopeful therapeutic strategy for ameliorating HCC pathogenesis from a variety of aetiologies including alcoholic hepatitis, fatty liver and viral hepatitis, by attacking cell death and tumor microenvironment (23). Meanwhile, numerous chemical agents and botanic drugs have been intensively tested as inhibitors for NLRP3 inflammasome and its constituents as effective modalities in prevention of HCC development from precedent hepatitis, fibrosis, and cirrhosis (24, 25). Nevertheless, a common drink, coffee, has long been overlooked in regard of the purpose mentioned above.

## Caffeine inhibits NLRP3 inflammasome activation

The capability of inhibiting NLRP3 inflammasome by caffeine has been revealed in several recent experimental cell and animal models. First of all, caffeine seems capable to protect the central nervous system from various insults by NLRP3 inflammasome inhibition. Caffeine is able to improve microglial polarization and long-term cognitive function in neonatal rats with hypoxic-ischemic white-matter damage through adenosine A2a receptor (A2aR)-mediated inhibition of NLRP3 inflammasome activation (26). Attenuation of microglia-mediated neuroinflammation in experimental autoimmune encephalomyelitis can also be achieved by caffeine with promotion of autophagy and inhibition of NLRP3 inflammasome activation in microglia (27). Sepsis-associated encephalopathy could be well protected by caffeine *via* inhibition of the uncoupling protein 2-mediated NLRP3 inflammasome pathway, leading to decreased neuronal apoptosis and mitochondrial dysfunction in astrocytes (28). In other systems, a study in neonatal mice discloses that caffeine can lower apoptosis of type 2 alveolar epithelial cells, preventing hypoxia-induced lung injury, through inhibition of NLRP3 inflammasome and NF-κB pathway (29). Another investigation in lipopolysaccharide-induced macrophages from human monocyte leukaemia cell line reveals that caffeine inhibits NLRP3 inflammasome activation, and thus decreasing secretion of IL-1β and IL-18, by suppressing mitogen-activated protein kinase/NF-κB signalling and A2aR-associated reactive oxygen species production (30). No wonder, it has been proposed that inhibition of inflammasome activation by caffeine with the consequent anti-inflammation and anti-pyroptosis effects probably could provide beneficial effects in fighting against SARS-CoV-2 infection (31) and ageing (32).

## A hypothesis for the mechanism of coffee’s chemopreventive effects on hepatocellular carcinoma

The integration of all the information above logically leads to a hypothesis that the positive effect of coffee in decreasing the risk of HCC derives from caffeine’s capability of inhibiting NLRP3 inflammasome activation. To put it another way, alleviating chronic inflammation process in the liver by drinking coffee can substantially lead to a reduced incidence of HCC. This speculation seems to be compatible with the facts that coffee consumption reduces not only the incidence of liver cancer but also risks of chronic liver disease and cirrhosis (33). It is hoped that the protective efficacy of caffeine against HCC could be demonstrated clearly by carefully designed animal experiments in the near future. Upon waiting definite research results, nonetheless, other possible mechanisms regarding caffeine’s beneficial influence on inflammation and carcinogenesis ought to be discussed as well.

## Discussion

As everybody may know, type 2 taste receptors (TAS2Rs), the bitter taste-sensing receptors, distribute widely in human organs and, once stimulated, have unidentified anticancer activity (34). It has been proved that several TAS2Rs exist in murine hepatocyte cell line (35). In addition to its presumed anti-inflammatory effects through acting on TAS2R and A2aR (and thus contributing to mitigating HCC pathogenesis) (36), caffeine probably also has unknown anticancer effects through activation of TAS2Rs. It is worthy to note that caffeine does have the capability of inhibiting human liver cancer cells in the laboratory (37). However, before going too quick to a conclusion, we have to recognize the fact that the inverse association with coffee consumption is less clear in caners arising from oesophagus, pancreas, kidneys, bladder, colorectum, ovaries, and prostate due to conflicting results or statistically nonsignificant data (38).

There are more than one thousand bioactive components in brewed coffee and the key beneficial compounds are attributed to polyphenolic agents, including caffeine, cafestol, kahweol, and chlorogenic acid (38). Although the diterpenes, cafestol and kahweol, have been demonstrated to have anti-inflammation and anti-carcinogenesis effects (39), they are present only in minimal amounts in instant and filtered coffee, which, however, seem also to have preventive effects on HCC (5). The chlorogenic acid, on the other hand, have been found to have the ability of inhibiting inflammasome NLRP3 activation in a variety of experimental models (40–42) and should therefore become another potential candidate of research interest in addition to caffeine in this regard.

Anyway, in accord with the hypothesis raised here, a lot of chemical agents and herb drugs besides caffeine have been examined in animal studies to see whether some of them could be used as the adequate medicine to attenuate inflammasome activity and slow the progress of alcohol-associated liver disease (43, 44), high-fat diet-associated liver disease (45–48), fulminant hepatitis (49), and acute liver injury (50). Although we highly suggested that caffeine be tested for similar objectives according to its ability of inhibiting NLRP3 inflammasome activation, caffeine has its own adverse effects when consumed too much, for example, increasing blood pressure, inducing anxiety, reducing skeletal-muscle insulin sensitivity, and decreasing infant birth weights (51). Moderate doses of coffee (40-200 mg per day) are thus recommended by health experts (52).

## Conclusions

Epidemiology data point out confidently that coffee drinking is inversely correlated with the incidence of HCC. Caffeine has been proved to be able to inhibit NLRP3 inflammasome activation in a series of cellular and animal studies. A hypothesis is raised here that the mechanism of coffee’s beneficial effects on prevention of HCC relies on the capability of caffeine in suppressing NLRP3 inflammasome activation and the ensuing inflammatory process, pyroptosis, hepatic fibrosis, and cirrhosis. Other mechanisms might exit also and the hypothesis needs further laboratory work and clinical trials to confirm. Having a habit of drinking coffee, of course, cannot guarantee a healthy liver without maintaining healthy life styles.

## Author contributions

FF alone conceptualized the idea, collected the literature, retrieved the database in the internet, and wrote the whole paper. The author confirms being the sole contributor of this work and has approved it for publication.

## Acknowledgments

The author likes to express his appreciation to the librarian Miss Wenyi Shaw, who works in the Ministry of Health and Welfare Changhua Hospital, Taiwan, for her help in retrieving scientific articles necessary for constructing the hypothesis in this paper.

## Conflict of interest

The author declares that the research was conducted in the absence of any commercial or financial relationships that could be construed as a potential conflict of interest.

## Publisher’s note

All claims expressed in this article are solely those of the authors and do not necessarily represent those of their affiliated organizations, or those of the publisher, the editors and the reviewers. Any product that may be evaluated in this article, or claim that may be made by its manufacturer, is not guaranteed or endorsed by the publisher.
